# Editorial: Targeting Myeloid Cells to Fight Cancer

**DOI:** 10.3389/fimmu.2019.02835

**Published:** 2019-12-03

**Authors:** Maija Hollmén, Wei Zheng, Jeffrey W. Pollard

**Affiliations:** ^1^MediCity Research Laboratory, University of Turku, Turku, Finland; ^2^Icahn School of Medicine at Mount Sinai, New York, NY, United States; ^3^MRC Centre for Reproductive Health, The University of Edinburgh, Edinburgh, United Kingdom; ^4^Department of Developmental and Molecular Biology, Albert Einstein College of Medicine, New York, NY, United States

**Keywords:** tumor microenvironment, monocyte, macrophage, neutrophil, immunosuppression, immunotherapy

Refractory and relapsed cancer patients often develop resistance mechanisms associated with an immunosuppressive tumor microenvironment (TME). Indeed, the TME is critically involved not only in tumor growth, invasion and dissemination, but in immune editing and resistance to different therapies both conventional and biological. Innate myeloid cells such as monocytes, macrophages, neutrophils, and a diverse set of cells, sometime called myeloid derived suppressor cells (MDSCs) may drive both local and systemic immunosuppression. These cells, however, possess novel molecules and functions that could be targeted to fight against cancer making them potential candidates for development of second-generation immunotherapy approaches. The aim of this article collection is to provide a comprehensive overview of the different myeloid subsets in the TME, and to describe recent developments and approaches targeting myeloid cells to enhance anti-tumor immunity and the clinical efficacy of standard-of-care cancer drugs.

## Heterogeneity of Myeloid Populations

Neutrophils exhibit diverse and sometimes contradictory roles in the TME. The review by Jeong et al. summarizes the regulation of neutrophils that determines the reciprocal dynamics between them and tumor cells. This continuous interaction has expanded to a formidably complicated network, including tumor types, tumor stages, subtypes of neutrophils, various signaling molecules mediating crosstalk, and spatial-temporal interactions with other cell types. This ever-growing complexity as reviewed by Jeong et al. has revealed how much more there is still to be learned to be able to achieve effective and consistent therapeutic effects based upon targeting neutrophils. This diversity is a point emphasized in the mini-review by Granot who suggests that simple binary classifications are insufficient and that the context defines neutrophil types and functions. Mackey et al. in their minireview address a particular aspect of the contextual regulation of neutrophils in the TME, i.e. neutrophil maturation outside the bone marrow. This atypical maturation and notably the promotion of immature neutrophils by tumors underlie many of the different responses of neutrophils to cancer.

Similar to neutrophils, monocytes also show significant diversity in the TME. In their review, Canè et al. support the notion of a “monocyte continuum” instead of classification of stepwise differences between the different subtypes. They also maintain that monocytes are more than precursors of macrophages and dendritic cells but also make a direct contribution to cancer development themselves. An additional feature of this review is the summary of current monocyte-targeting drugs and their mechanisms of actions. Along these lines, Mengos et al. describes the documented clinical findings on a CD14^+^HLA-DR^lo/neg^ monocyte population that has emerged as an important mediator of tumor-induced immunosuppression. Different from MDSCs, these cells arise from the regular circulating monocyte pool, which lose HLA-DR expression and get deactivated after an acute or chronic inflammatory trigger leading to immune paralysis. The immunosuppressive monocytes can negatively affect responses to immune checkpoint inhibitors and could be potentially used as biomarkers to understand disparate responses to immune activating drugs, in addition to their potential targeting to increase immunotherapy responses.

## Seeing is Believing

Cellular processes as analyzed by tissue sections at a certain time point may be missed, impossible to obtain or even misinterpreted. With new technologies, intravital optical imaging is providing an unprecedented opportunity to look at cancer cells and stromal cells in their native environment and thus yields arguably the most reliable results in interpreting the TME. Laviron et al. examine the pros and cons of intravital imaging as compared to other approaches, such as cell sorting and sequencing, and suggest that an integrative approach offers the most valuable information.

## Compounds Affecting Myeloid Functions in the TME

The mode of action of several FDA approved drugs such as bisphosphonates, trabectedin, imatinib, and sunitinib has been shown to involve the modulation of macrophage functions in addition to their respective targets. Whether their macrophage targeting effects are significantly contributing to drug efficacy remains to be investigated. In their article Tanita et al. shed light on the mode of action of bexarotene, a third-generation retinoid that has been used for decades in the treatment of both early and advanced cutaneous T-cell lymphomas (CTCL). The authors show that bexarotene decreases CCL22 production by CD163^+^ macrophages in CTCL patients. Since CCL22 attracts CCR4^+^ lymphocytes, such as Tregs, Th2 cells, and also CTCL cells to support tumor progression, this study proposes the potential of targeting tumor associated macrophages (TAMs) in CTCL for improved patient outcome (Tanita et al.)

Another interesting concept is reported by Willebrand et al. where they studied the effect of high salt intake on the functional modulation of MDSCs in the TME. Excess salt intake has been previously shown to balance various innate and adaptive immune cells toward a pro-inflammatory state, and believed to be associated with several autoimmune disorders. Therefore, an interesting question that the authors raised was whether high salt conditions can promote anti-tumor immunity to inhibit tumor growth? Surprisingly, the authors find that, while high-salt diet altered T cell populations, the delay in tumor growth was largely mediated by an impairment in MDSC suppressive functions (Willebrand et al.) Thus, high salt-induced molecular changes could be potentially utilized during immunotherapy.

## Possibilities to Target Myeloid Cells For Improved Cancer Care

As a general assumption, macrophages are mostly considered to have an unfavorable role in cancer since they are effective suppressors of anti-tumor immune responses and can contribute to tumor progression in multiple ways. Tumor growth is often compared to the wound healing process and is metaphorically described as a “wound that does not heal.” Hua and Bergers focus on two common components in these two intricately related processes—blood vessels and immune cells. They explain how tumor cells hijack the immunosuppressive and angiogenic programs that occur during the resolution phase of wound healing toward their own ends. The authors thus suggest targeting both myeloid cells and angiogenesis to revert the hijacked wound-healing process.

In a mini review Bercovici et al. discuss the plasticity of macrophages and focus on macrophage regulated intratumoral T cell migration and activation. The authors emphasize that T cell focused treatments should be systematically replaced by rational combination treatments stimulating both innate and adaptive arms of the immune system. When appropriately stimulated macrophages can effectively induce anti-tumor responses in cooperation with T cells. To date, only a few myeloid cell targeting strategies under clinical development have yielded promising results and many have been terminated due to toxicities. In their review Jahchan et al., indicate the potential of targeting TAMs in cancer immunotherapy. They make a persuasive case for such approaches through the targeting of particular functions of TAMs or by altering their differentiation to anti-tumoral responses either directly or through the enhancement of immunotherapy. They also provide an authoritative update of all current clinical trials using these approaches.

As shown by this collection of articles, a tremendous diversity of myeloid cells orchestrate tumor-stromal and stromal-stromal interactions to regulate the final outcome of the disease. Elucidation of the detailed mechanisms and systemic rational evaluation of therapeutic targeting of these cells may be the key to fighting cancer.

## Author Contributions

All authors listed have made a substantial, direct and intellectual contribution to the work, and approved it for publication.

### Conflict of Interest

The authors declare that the research was conducted in the absence of any commercial or financial relationships that could be construed as a potential conflict of interest.

